# microRNA-1271-5p/TIAM1 suppresses the progression of ovarian cancer through inactivating Notch signaling pathway

**DOI:** 10.1186/s13048-020-00720-w

**Published:** 2020-09-18

**Authors:** Feng-Juan Han, Jia Li, Ying Shen, Ying Guo, Yi-Chao Liu, Yang Yu, Jia-Yue Xu, Shao-Xuan Liu, Yan-Hong Wang

**Affiliations:** 1 Department of Gynecology, The First Affiliated Hospital of Heilongjiang University of Traditional Chinese Medicine, Harbin, 150040 Heilongjiang China; 2 Heilongjiang University of Traditional Chinese Medicine, First Clinical Medical College, Harbin, 150040 Heilongjiang China; 3 Department of Chinese medicine, Harbin Institute of Technology Hospital, Harbin, 150006 Heilongjiang China; 4 Heilongjiang University of Traditional Chinese Medicine, College of Pharmacy, No. 24, Heping Road, Xiangfang District, Harbin, 150040 Heilongjiang Province China

**Keywords:** miR-1271-5p, Ovarian cancer, Prognosis, Cell behavior, Notch pathway

## Abstract

**Objective:**

Ovarian cancer (OC) has been regarded as the most malignant gynecological neoplasm and often confers grave outcomes owing to the frequent metastasis and high recurrence. A previous study has demonstrated that miR-1271-5p is implicated in OC progression, however, the possible mechanism of it remains unknown. The purpose of this investigation was to explore how miR-1271-5p regulates the progression of OC.

**Methods:**

Gene Expression Omnibus (GEO) and The Cancer Genome Atlas (TCGA) databases were employed to analyze the differentially expressed miRNAs or genes as well as their corresponding prognostic values. miR-1271-5p expression in OC cells was examined by qRT-PCR. Cell counting kit 8 (CCK-8), colony formation, and transwell tests were conducted to evaluate the proliferation, migration and invasion potentials. Bioinformatics prediction and luciferase activity analysis were utilized to predict and verify the target gene of miR-1271-5p. Western blot assay was carried out to measure protein expression.

**Results:**

miR-1271-5p was significantly decreased in OC and its down-regulation was associated with the grave outcome of OC patients. Upregulation of miR-1271-5p inhibited cell viability, but miR-1271-5p knockdown promoted the proliferation of OC cells. TIAM1 was a direct target gene of miR-1271-5p and expressed in OC tissues at higher level. High expression of TIAM1 induced the poorer prognosis of patients with OC. Further functional analyses showed that the suppressive role of miR-1271-5p on OC cell malignant behaviors was overturned by the upregulation of TIAM1. The protein levels of Cyclin D1, HES1, NOTCH and NUMB were remarkably changed due to the abnormal expression of miR-1271-5p and TIAM1.

**Conclusion:**

To sum up, miR-1271-5p inhibits proliferation, invasion and migration of OC cells by directly repressing TIAM1 to inactivate the Notch signaling pathway, which provides an alternative therapeutic candidate for the advancement of OC treatment.

## Background

Ovarian cancer (OC) is a principal reason of cancer death from gynecological malignancy, which accounts for 2.4–6.5% of tumors in females [1]. The status of OC in reproductive system cancers of women is second only to cervical cancer and endometrial cancer [2]. The surgery and radio-chemotherapy are the accustomed therapeutic strategies for OC [3]. Statistical data reveal that the five-year survival rate of all OC patients is only 47%, even less than 20% in advanced stage patients [4]. Therefore, investigating the potential indicators for the early diagnosis and identifying efficacious targets are urgently required to be developed for OC patients.

miRNAs are endogenous short RNAs composed of 21–25 nucleotides involved in tumorigenesis [5, 6]. Several studies suggest the crucial effect of miRNAs in OC development. miR-212-3p functions as a key target and suppresses the progression of high-grade serous OC via interacting with MAP 3 K3 [7]. The cyst formation and mode of OC spread are governed by miR-200 family [8]. Paliwal et al. illustrated that miR-22 and miR-21 are wonderful diagnostic indicators for OC early diagnosis [9]. miR-30b-3p as an anti-tumor gene in OC represses the migration and invasion of cells via down-regulating the CTHRC1 gene [10]. Among the massed miRNAs, miR-1271-5p is expressed at abnormal levels in many human cancers and participates in the tumorigenesis [11, 12]. Of note, a ten-miRNA signature including miR-1271-5p has been identified from the genome-wide miRNA expression profiling in OC [13]. This suggests that miR-1271-5p maybe a promising therapeutic molecule to treat OC. However, the potential effect of miR-1271-5p in human OC as well as its underlying molecular mechanism has been poorly understood.

TIAM1 (T cell lymphoma invasion and metastasis 1) is initially found in mice T lymphoma cells and considered as a metastasis-related gene [14]. Extensive evidences have illustrated that TIAM1, as a guanine nucleotide exchange factor, is involved in the invasion and metastasis of various human tumors, such as gastric cancer [15], prostate cancer [16] and breast cancer [17]. A previous report uncovered that TIAM1 expression is closely related with the grade and prognosis in OC and might be helpful for the management of clinic [18]. TIAM1 has also been indicated to facilitate the growth and invasion of OC cells through negatively being regulated by miR-22, miR-183 and miR-31 [19]. Despite that, the relevance between miR-1271-5p and TIAM1 in OC still remain elusive.

Recently, Notch signaling pathway has aroused more attention for carcinogenesis, which acts as an evolutionarily conserved pathway involved in the regulation of multiple biological processes, including proliferation, apoptosis, invasion and differentiation [20, 21]. The Notch family is composed of four receptors and five ligands [22]. Notably, Notch pathway has been reported to affect the progression of different kinds of cancers [23]. TIAM1 was positively related with the Notch signaling pathway. Furthermore, an investigation indicated that a crosstalk exists between miRNAs and Notch pathway in multiple malignancies [24].

Herein, this work aimed to evaluate the biological role of miR-1271-5p in OC, so as to decipher the prospective mechanism and its prognostic value. All results showed that miR-1271-5p might exert a suppressive role on the progression of OC, and we also discovered that miR-1271-5p/TIAM1 axis regulates the development of OC through mediating the Notch signaling pathway. These findings can advance our understanding of OC progression and potential treatment options for OC.

## Methods

### Tissue samples

Gene Expression Omnibus (GEO; https://www.ncbi.nlm.nih.gov/geo/) is an open public genomics repository and used to help researchers inquire and download gene expression profiles. The data set GSE119055 derived from GEO was prepared to show the difference of miR-1271-5p expression level in normal controls (*n* = 3) and OC tissue samples (*n* = 6). Expression of TIAM1 in OC and its prognostic value were analyzed using TCGA-OC cohort (https://cancergenome.nih.gov/) that included 378 OC tissue samples. The normal specimens (*n* = 88) were provided by Genotype-Tissue Expression (GTEx) project (http://commonfund.nih.gov/GTEx/).

### Cell culture and transfection

Human normal control cell line IOSE80 (SXBIO Co., Ltd., Shanghai, China) and human OC cell lines (SK-OV-3, Cell Biology of the Chinese Academy of Sciences, Shanghai, China; OVCAR-3, American Type Culture Collection, Manassas, VA, USA) were incubated in Dulbecco’s Modified Eagle’s Medium (DMEM, Gibco, Grand Island, NY, USA) supplemented with 10% fetal bovine serum (FBS, Gibco) and specific antibiotics (100 U/mL penicillin and 0.1 mg/mL streptomycin) at 37 °C with 5% CO_2_.

miR-1271-5p mimic (5′-CUU GGC ACC UAG CAA GCA CUC A-3′), inhibitor (5′-UGA GUG CUU GCU AGG UGC CAA G-3′) and scrambled miR-negative control (NC; 5′-UCA CAA CCU CCU AGA AAG AGU AGA-3′); small inference RNAs against TIAM1 (si-TIAM1#1 and si-TIAM1#2) and corresponding NC (si-con) were synthesized by Genepharma Co., Ltd. (Shanghai, China). Full length TIAM1 was cloned into the pcDNA3.1 vector and the resultant was named as pcDNA3.1-TIAM1 (TIAM1). Its negative control was named as pcDNA3.1-empty vector (vector). Cells were transfected with above agents using Lipofectamine2000 (Life Technologies, USA). Twenty-four hours later, the transfection efficiency was measured with qRT-PCR test.

### RNA isolation and qRT-PCR

qRT-PCR was performed to measure the expression of miR-1271-5p and TIAM1 in OC cells. Firstly, TRIZOL solution was applied to isolate the total RNA of transfected OVCAR-3 and SK-OV-3 cells which was then reverse transcribed into cDNA by the PrimeScript RT reagent Kit (Takara, Dalian, China). SYBR Premix Ex Taq II was applied for the performance of qRT-PCR on ABI 7500 PCR system (Foster, CA, USA). miR-1271-5p and TIAM1 expression was normalized to U6 or GAPDH, respectively. Quantification of miR-1271-5p and TIAM1 expression was determined by 2^-△△Ct^ method. Primers of this present study are revealed in Table 1.

**Table 1:** Sequences of primers used in this study

Primer name	Sequence
miR-1271-5p	F: 5′- TTGGCACCTAGCAAGCA −3′
	R: 5′-GAACATGTCTGCGTATCTC-3′
U6	F: 5′-CGCAAATTCGTGAAGCGTTC-3′
	R: 5′-TTTGCGTGTCATCCTTGCG −3′
TIAM1	F: 5′- AAATCACACGGCGACCTGTCGT −3′
	R: 5′- ATGGCATCCTGAAGCCTCATCC − 3′
GAPDH	F: 5′- GTCAAGGCTGAGAACGGGAA − 3′
	R: 5′- AGTGATGGCATGGACTGTGG − 3’

### Protein extraction and western blot

Western blotting was used to detect the expression levels of indicated proteins. After 24 h transfection, RIPA buffer supplemented with protease inhibitor PMSF was utilized to isolate proteins from OVCAR-3 and SK-OV-3 cells. The bicinchoninic acid (BCA) quantification kit (MultiSciences Biotech Co., Ltd., Shanghai, China) was applied to quantify the concentration of proteins. Separated proteins were firstly denatured at 95 °C for 5 min. Equal quantities of denatured protein (20 μg) were loaded in 12% SDS-PAGE and electrophoretically transferred onto the PVDF membranes. PVDF membranes were sealed with 5% skimmed milk for 1 h at 37 °C, and subsequently incubated with specific primary antibodies at 4 °C overnight and HRP-tagged secondary antibody at 37 °C for 1 h. Following washing membranes with PBST, the protein signals were probed wit the ECL reagent. The protein signal was analyzed by ImageJ software.

### Cell viability analysis

The proliferation capacity of OVCAR-3 and SK-OV-3 cells was elevated using CCK-8 assay. Briefly, transfected cells (1000 cells/well) were seeded into a 96-well plate and cultured at 37 °C with 5% CO_2_ for 0, 24, 48 and 72 h. Then, 10 μL of CCK-8 solution was put into every well for additional 1.5 h incubation. Afterwards, a microplate reader was utilized to detect the OD values under 450 nm to assess the cell viability at predefined time points.

### Colony formation

The clonogenicity of OC cells was measured by colony formation experiments. After specific transfection, 1000 cells per well were inoculated into the six-well plates and cultured in DMEM medium with 10% FBS at 37 °C with 5% CO_2_ for two weeks. Next, colonies observed by naked eyes were fixed in 4% paraformaldehyde and dyed in 0.1% crystal violet for 30 min, respectively. Washed the staining reagent gently using running water and artificially counted the number of colonies with more than 50 cells under a microscope. Three plates were seeded for each cell line and this experiment was repeated on three different days.

### Transwell test

The migration and invasion capabilities of OVCAR-3 and SK-OV-3 cells were examined using a transwell test. The upper surface of transwell chambers pre-coated with Matrigel was employed to detect invasion ability, and the migration capacity detection required no Matrigel. After 24 h transfection, OC cells (1 × 10^5^) were placed in the top chamber with 100 μL serum-free medium; simultaneously, culture medium (500 μL) was added into the bottom chamber. Followed by overnight incubation, cells remain on the upper surface were removed using the cotton swabs and the migrated or invaded cells in the lower transwell chamber were fixed in 4% paraformaldehyde and dyed with 0.1% crystal violet for 30 min, respectively. Cells were washed using PBS for three times in triplicates. Five random fields were picked and the numbers of migrated or invaded cells were counted under a microscope.

### Dual-luciferase activity assay

In order to verify the candidate targets, luciferase activity assay was implemented. The specific dual-luciferase reporter vectors (wild type-TIAM1 and mutant-TIAM1) were synthesized and transfected into OC cells by using Lipofectamine2000 according to the guidelines of manufacturer. Forty-eight hours later, transfected cells were collected for the measurement of the luciferase activity by a dual-luciferase reporter assay kit (Promega, Madison, WI, USA).

### Statistical analysis

All experiments were done in triplicates, and each test was repeated three times. Results were statistically analyzed using SPSS22.0 software and GraphPad Prime 8.0, and exhibited as mean ± SD. Student’s t-test and ANOVA was performed to compare the differences in two or more groups. Kaplan-Meier method was utilized to plot the overall survival curve. The relationship between miR-1271-5p and TIAM1 in OC was identified using Pearson’s correlation analysis. Gene set enrichment analysis (GSEA) enrichment was performed with GSEA (version2.2.2, http://www.broadinstitute.org/gsea). *P* < 0.05 was regarded as significance.

## Results

### Downregulation of miR-1271-5p in OC is related with unfavorable prognosis and elevates cell viability

**Fig. 1:**
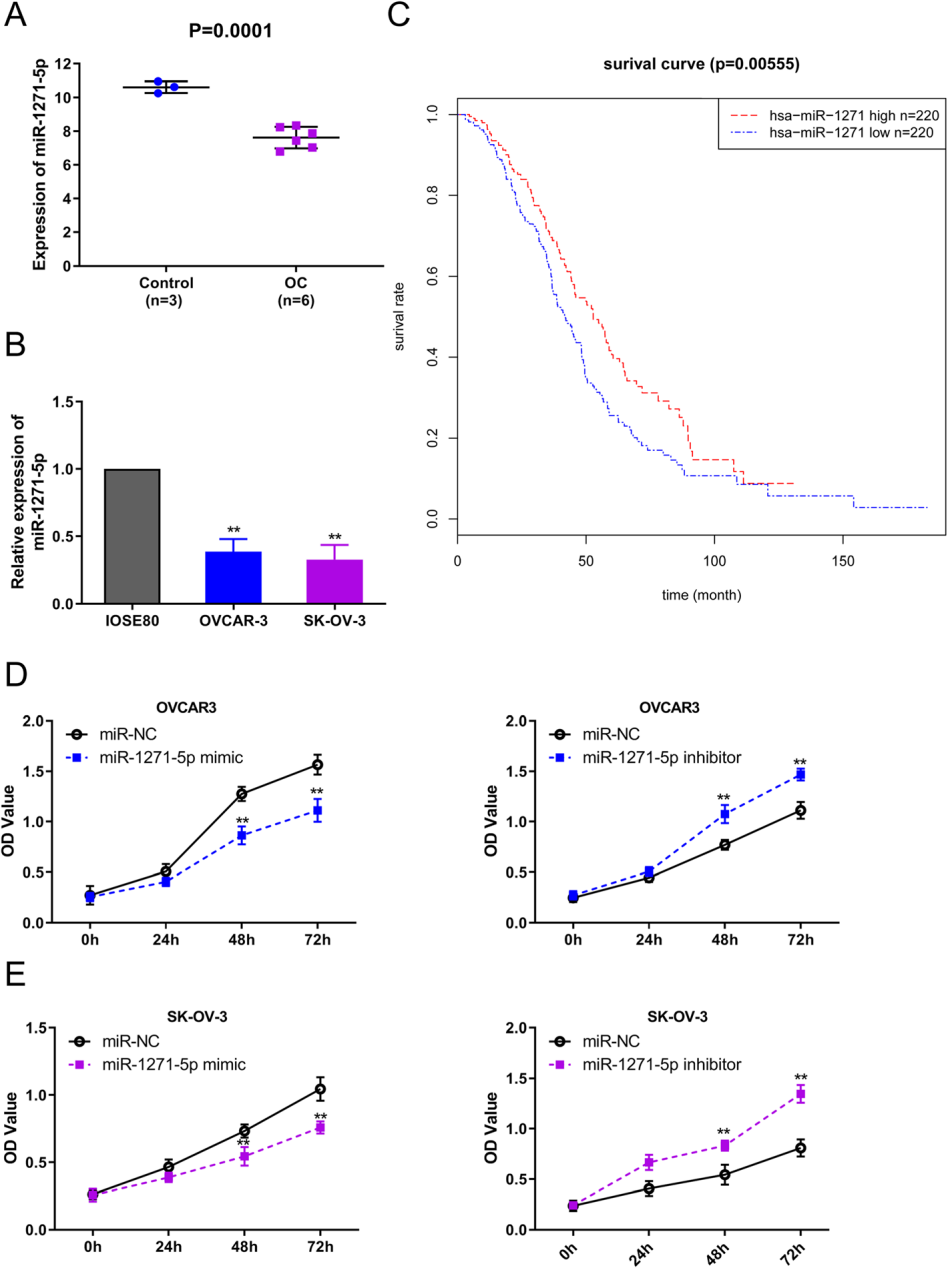
miR-1271-5p was decreased in OC and affected the prognosis of patients with OC. **a** The expression level of miR-1271-5p in normal control (*n* = 3) and OC tissue samples (*n* = 6) based on the GSE119055 array, *P* = 0.0001. **b** RT-PCR assay was implemented to detect the expression level of miR-1271-5p in different cell lines, ^**^P < 0.01 versus IOSE80 cell line. **c** Kaplan-Meier curve of miR-1271-5p expression in OC of TCGA-OC cohort, *P* = 0.00555. **d-e** The proliferation capacity of OVCAR-3 and SK-OV-3 cells were measured using CCK-8 assay, ^**^P < 0.01 versus miR-NC group

In order to explore the possible therapeutic targets that exert crucial effects in OC development, we firstly screened the differential expression miRNAs in GSE119055 by using R package “limma” with FDR < 0.05 and | log (FC) | ≥ 2. Analysis showed that a total of 25 miRNAs were expressed with lower levels in OC tissue samples compared with normal controls. Subsequently, the prognostic power of these 25 down-regulated miRNAs was analyzed with the threshold of *P* < 0.05 and FDR < 0.05 based on TCGA-OC cohort. Finally, three miRNAs including miR-1271-5p, miR-501-3p and miR-500a-3p were identified differential expression miRNAs with potential prognostic value in OC. Notably, miR-1271-5p ranked the second lowest based on the fold change. Thus, based on the comprehensive literature analysis, miR-1271-5p was picked for future experimental analysis. Figure 1a and b showed that miR-1271-5p was declined in OC tissues (*P* = 0.0001) and cell lines (^**^P < 0.01). On the basis of the TCGA-OC cohort, OC clinical samples were assigned into high-miR-1271-5p group and low-miR-1271-5p group owing to the median value of miR-1271-5p expression. OC patients with a lower level of miR-1271-5p had poorer outcomes compared to that with high miR-1271-5p level (Fig. 1c, *P* = 0.00555). CCK-8 assay was carried out to measure the proliferation potential of OC cells (Fig. 1d and e, ^**^P < 0.01). Results disclosed that the inhibition of miR-1271-5p expression promoted the proliferation, whereas overexpression of miR1271-5p repressed cell viability, in both OVCAR-3 and SK-OV-3 cells. These data indicated that miR-1271-5p might act as a suppressive factor in OC development.

### TIAM1 is a direct target of miR-1271-5p

**Fig. 2:**
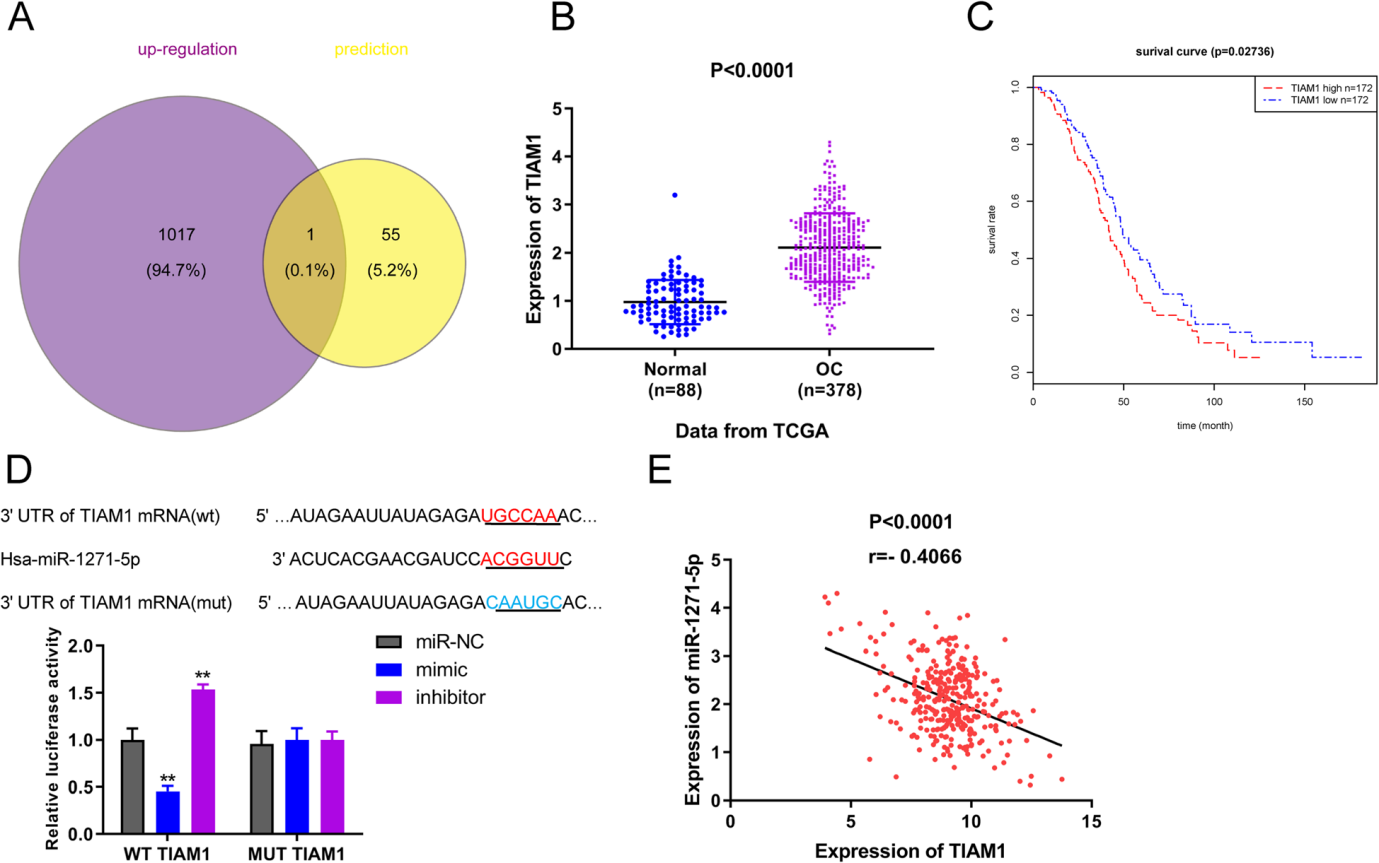
miR-1271-5p directly target TIAM1 in OC. **a** Venn diagram indicated that TIAM1 might be a candidate target of miR-1271-5p. Violet represents upregulated expression genes in OC; yellow represents putative target genes of miR-1271-5p. **b** TIAM1 was expressed at higher levels in OC samples (*n* = 378) compared with normal cases (*n* = 88) on the basis of TCGA database, *P* < 0.0001. **c** Overall survival curve of TIAM1 in OC, *P* = 0.02736. **d** The correlation between miR-1271-5p and TIAM1 was testified using luciferase reporter gene assay, ^**^P < 0.01 versus miR-NC group. (E) Pearson’s correlation analysis was applied to evaluate the relationship between miR-1271-5p expression and TIAM1 expression, *P* < 0.0001, r = − 0.4066

**Fig. 3:**
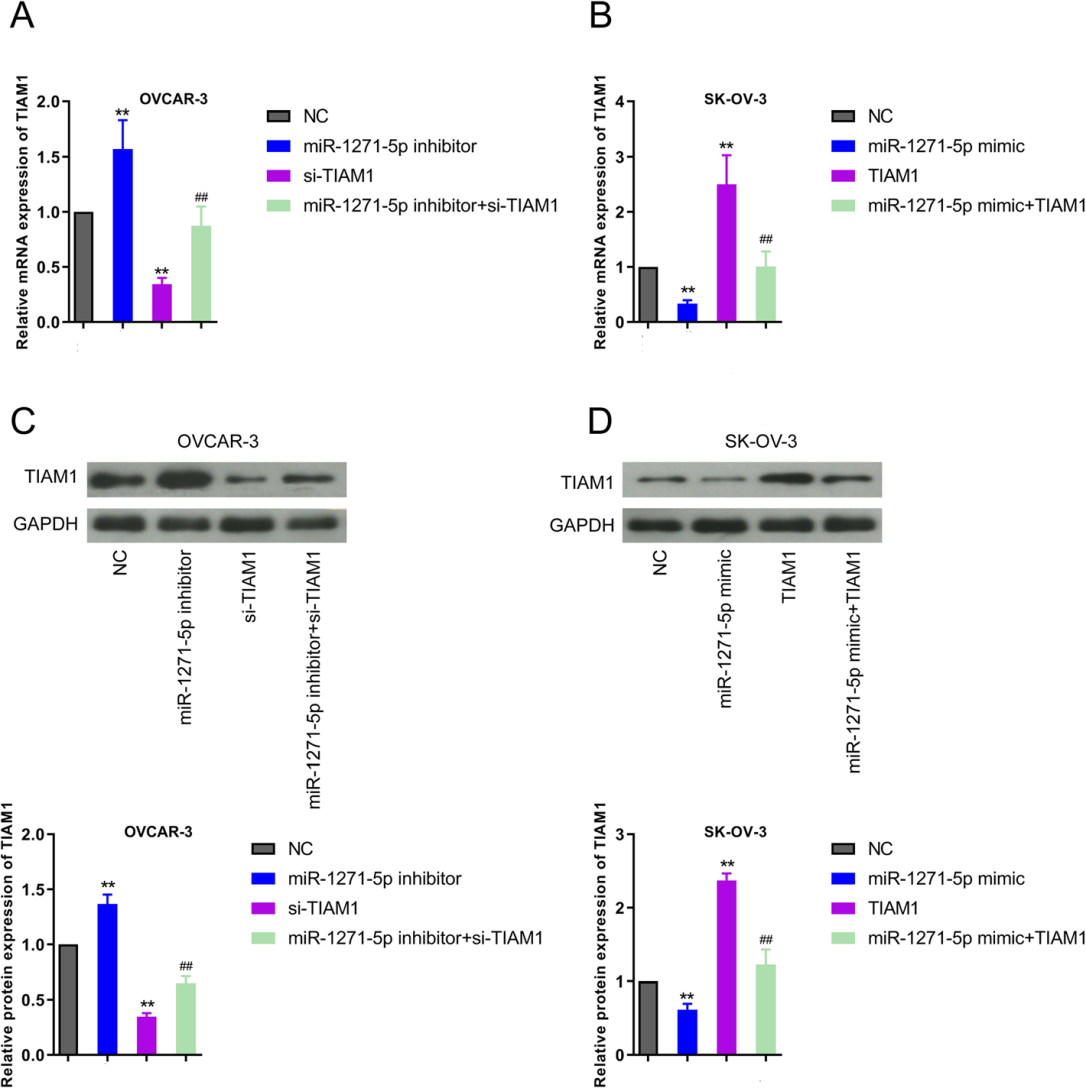
TIAM1 expression was negatively regulated by miR-1271-5p. **a-b** Relative mRNA expression of TIAM1 was assessed by qRT-PCR in OVCAR-3 and SK-OV-3 cells after different transfections, including inhibitor/mimic, si-TIAM1/TIAM1, inhibitor+si-TIAM1 or mimic+TIAM1, ^**^P < 0.01 versus NC group, ^##^*P* < 0.01 versus inhibitor or mimic group, ^&&^*P* < 0.01 versus si-TIAM1 or TIAM1 group. **c-d** After above transfections, western blot experiment was performed to measure the protein expression level of TIAM1 in OVCAR-3 and SK-OV-3 cells. The gray values of protein bands were quantified. ^**^P < 0.01 versus NC group, ^##^*P* < 0.01 versus inhibitor or mimic group, ^&&^*P* < 0.01 versus si-TIAM1 or TIAM1 group

To investigate the target gene of miR-1271-5p in OC, we firstly clustered the TCGA-OC cohort and normal specimens from GTE_X_ database to screen the differentially expression genes in OC, with FDR < 0.05 and |logFC| > 2. A total of 2254 differentially expression genes were obtained, including 1018 upregulated genes and 1236 down-regulated genes. Target prediction tool suggested that there were 56 putative targets of miR-1271-5p in OC. A common gene TIAM1 was ultimately obtained after intersecting upregulated genes and candidate target genes (Fig. 2a). Compared with normal samples, TIAM1 was expressed at higher levels in OC tissues (Fig. 2b, *P* < 0.001). The survival curve showed that high expression of TIAM1 was related with unfavorable prognosis compared with OC patients carrying low level of TIAM1 (Fig. 2c, *P* = 0.02736). Figure 2d manifested the binding site between miR-1271-5p and TIAM1. Results of luciferase reporter gene analysis demonstrated that the transfection of miR-1271-5p mimic attenuated the luciferase activity of WT TIAM1 group, whilst miR-1271-5p inhibitor markedly increased the luciferase activity, in both OVCAR-3 and SK-OV-3 cells. Simultaneously, in the OVCAR-3 and SK-OV-3 cells, the transfection of miR-1271-5p mimic or inhibitor exerted no impacts on the luciferase activity of MUT TIAM1 group (Fig. 2d, ^**^P < 0.01). In addition, Pearson’s correlation analysis confirmed that there was a negative relationship between miR-1271-5p and TIAM1 expression in OC (Fig. 2e, *P* < 0.0001, r = − 0.4066). qRT-PCR and western blot experiments verified the correlation between miR-1271-5p and TIAM1 expression in OC cells (Fig. 3, ^**^P < 0.01, ^##^*P* < 0.01, ^&&^*P* < 0.01). In OVCAR-3 cells, miR-1271-5p inhibitor transfection increased the expression of TIAM1, whilst the addition of si-TIAM1 reversed the effect of miR-1271-5p inhibitor on TIAM1 expression (Fig. 3a and c, ^**^P < 0.01, ^##^*P* < 0.01, ^&&^*P* < 0.01). By contrast, miR-1271-5p enhancement inhibited TIAM1 expression, which was rescued by the transfection of pcDNA3.1-TIAM1 in SK-OV-3 cells (Fig. 3b and d, ^**^P < 0.01, ^##^*P* < 0.01, ^&&^*P* < 0.01). All observations uncovered that miR-1271-5p can directly target TIAM1 and negatively regulated the expression of TIAM1 in OC.

### miR-1271-5p suppresses the proliferation, migration and invasion capacities of OC cells via targeting TIAM1

**Fig. 4:**
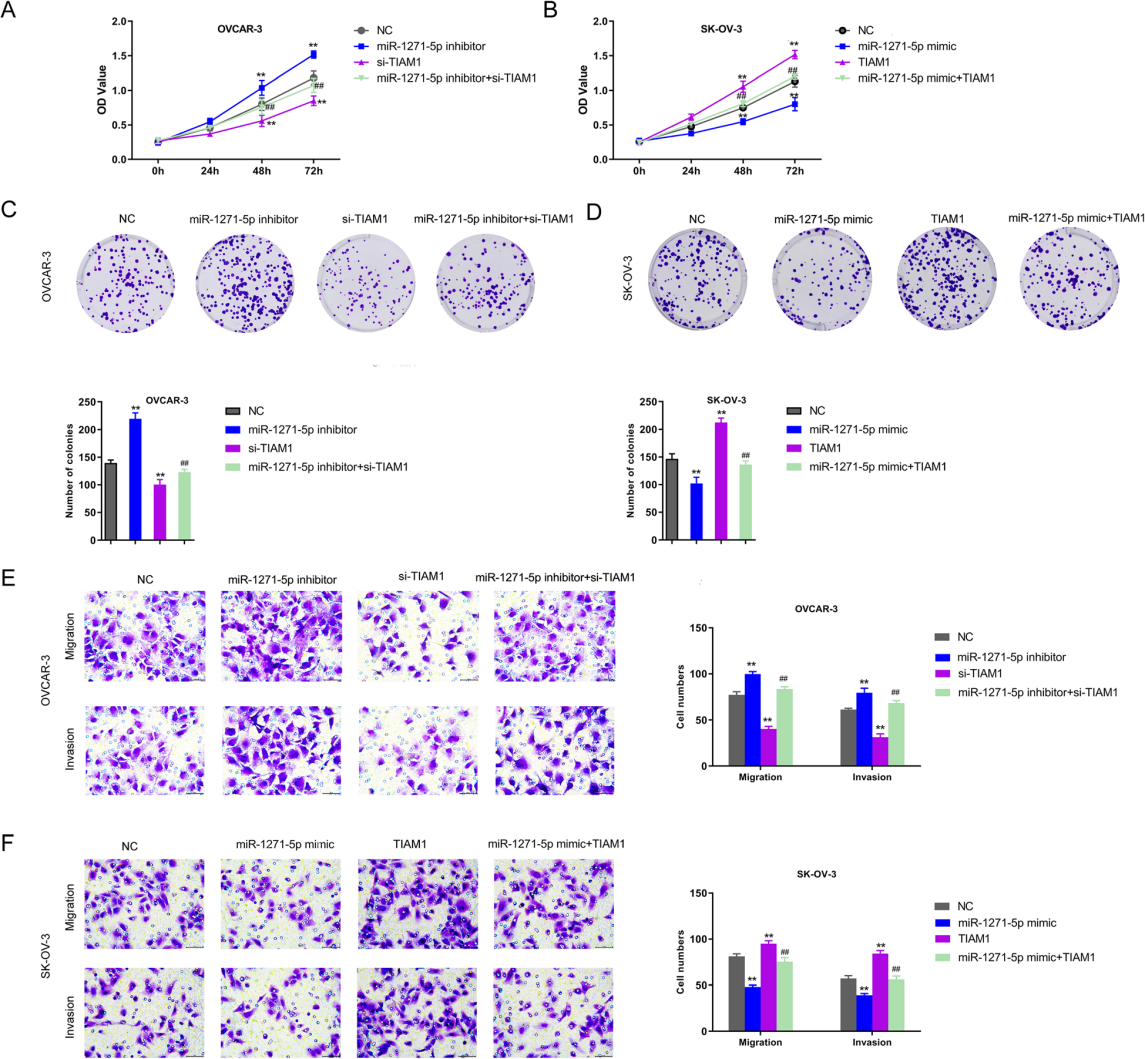
miR-1271-5p inhibited the development of OC through downregulating TIAM1. **a-b** The cell viability of OVCAR-3 and SK-OV-3 cells was detected by CCK-8 analysis, ^**^P < 0.01 versus NC group, ^##^*P* < 0.01 versus inhibitor or mimic group, ^&&^*P* < 0.01 versus si-TIAM1 or TIAM1 group. **c-d** A colony formation test was conducted to explore the clongenic ability of OVCAR-3 and SK-OV-3 cells. The number of colonies was counted, ^**^P < 0.01 versus NC group, ^##^*P* < 0.01 versus inhibitor or mimic group, ^&&^*P* < 0.01 versus si-TIAM1 or TIAM1 group. **e-f** Transwell migration and invasion experiments were used to investigate the migration and invasion capabilities of OVCAR-3 and SK-OV-3 cells, ^**^P < 0.01 versus NC group, ^##^*P* < 0.01 versus inhibitor or mimic group, ^&&^*P* < 0.01 versus si-TIAM1 or TIAM1 group

To in-depth assess the biological role of miR-1271-5p/TIAM1 in OC cellular malignant behaviors, OVCAR-3 cells were transfected with miR-1271-5p inhibitor, si-TIAM1 and miR-1271-5p inhibitor + si-TIAM1; SK-OV-3 cells were transfected with miR-1271-5p mimic, TIAM1 and miR-1271-5p mimic + TIAM1. CCK-8 results revealed that silencing TIAM1 suppressed the OVCAR-3 cell proliferation, and TIAM1 enhancement promoted cell viability of SK-OV-3 cells. Co-transfection of inhibitor + si-TIAM1 or mimic + TIAM1 was enable to overturn the individual effect induced by inhibitor, si-TIAM1 or mimic, TIAM1 on cell proliferation (Fig. 4a and b, ^**^P < 0.01, ^##^*P* < 0.01, ^&&^*P* < 0.01). Moreover, the colony formation test determined that the TIAM1 knockdown could abolish the elevating effect of miR-1271-5pinhibitor on clonogenic ability in OVCAR-3 cells, and upregulation of TIAM1 could recover the miR-1271-5p-induced suppressive role on colony formation of SK-OV-3 cells (Fig. 4c and d, ^**^P < 0.01, ^##^*P* < 0.01, ^&&^*P* < 0.01). The consistent results were presented in transwell migration and invasion analyses (Fig. 4e and f, ^**^P < 0.01, ^##^*P* < 0.01, ^&&^*P* < 0.01). To summarize, all data illustrated that miR-1271-5p might function as a tumor-suppressive factor in OC through targeting TIAM1.

### miR-1271-5p/TIAM1 axis regulates the activity of notch signaling pathway in OC

**Fig. 5:**
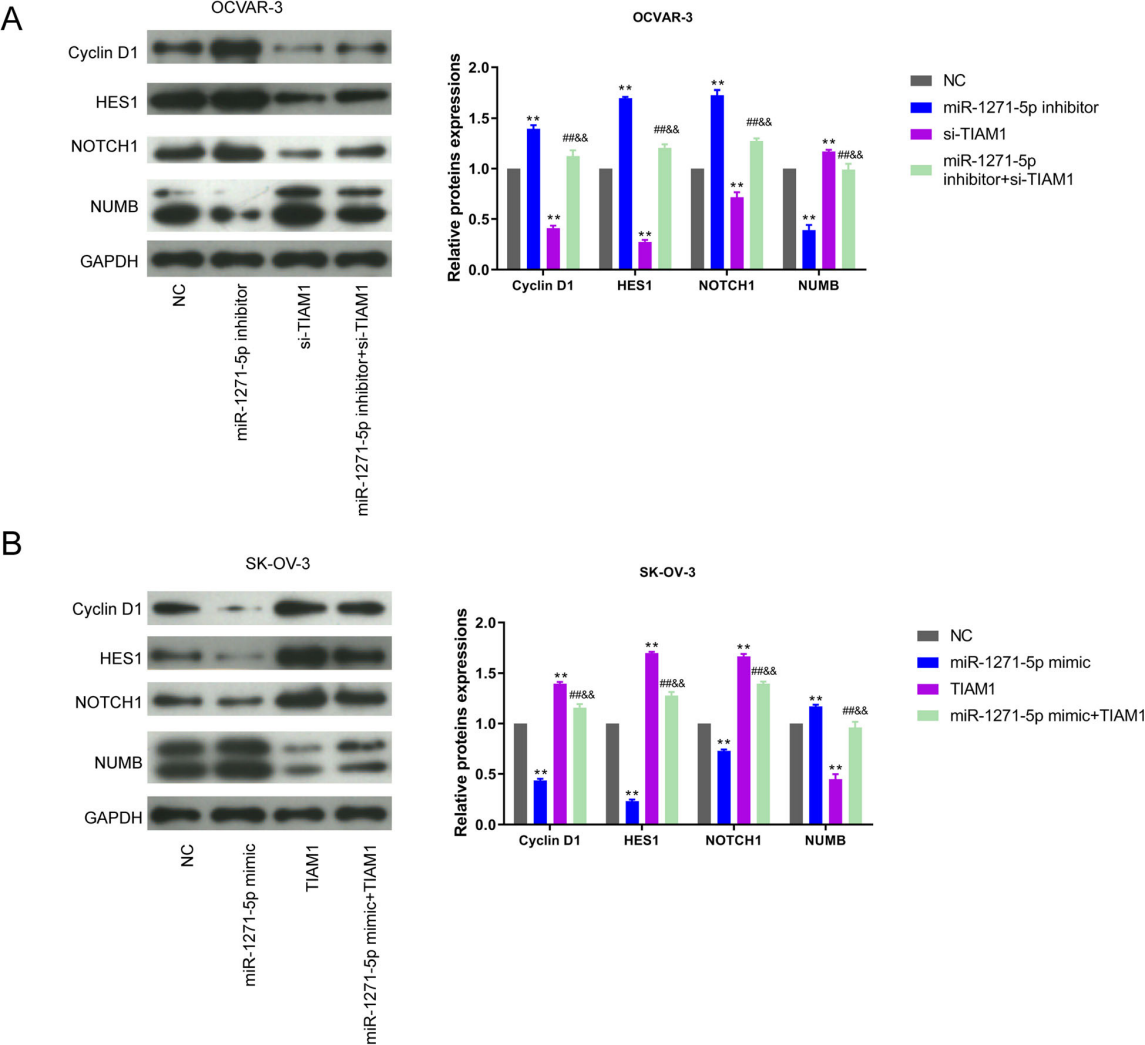
Notch signaling pathway was involved in the regulation of miR-1271-5p/TIAM1 in OC development. **a-b** The levels of Cyclin D1, HES1, NOTCH and NUMB were tested by western blot in transfected OVCAR-3 and SK-OV-3 cells. The quantitative of protein intensity were also showed in histograms, ^**^P < 0.01 versus NC group, ^##^*P* < 0.01 versus inhibitor or mimic group, ^&&^*P* < 0.01 versus si-TIAM1 or TIAM1 group

To explore how miR-1271-5p/TIAM1 axis regulates the progression of OC, GSEA enrichment analysis was performed and disclosed that TIAM1 was enriched in Notch signaling pathway. Next, we conducted western blot experiment to examine the effect of miR-1271-5p/TIAM1 axis on the Notch pathway. Results of western blot were shown in Fig. 5. As exhibited in Fig. 5a, miR-1271-5p inhibitor induced a increase level of Cyclin D1, HES1, and NOTCH, while declined the protein level of NUMB in OCVAR-3 cells. On the contrary, silencing TIAM1 attenuated the proteins levels of Cyclin D1, HES1, and NOTCH, elevated NUMB level. Interestingly, co-transfection miR-1271-5p inhibitor and si-TIAM1 recovered these proteins levels that significantly changed by miR-1271-5p inhibitor or si-TIAM1 (Fig. 5a, ^**^P < 0.01, ^##^*P* < 0.01, ^&&^*P* < 0.01). On the other hand, the results in SK-OV-3 cells revealed that overexpression of miR-1271-5p led to a lower level of Cyclin D1, HES1, and NOTCH, whereas NUMB was markedly increased. The opposite tendency of these proteins was caused by TIAM1 enhancement. Co-transfection of miR-1271-5p mimic+TIAM1 remarkably restored the expression levels of Cyclin D1, HES1, NOTCH, and NUMB to normal levels (Fig. 5b, ^**^P < 0.01, ^##^*P* < 0.01, ^&&^*P* < 0.01). Taken together, miR-1271-5p/TIAM1 axis regulates the progression of OC via mediating the activity of Notch signaling pathway.

## Discussion

Former studies have demonstrated the important regulatory role of miR-1271-5p in numerous cancers. For instance, miR-1271-5p can repress cell viability and promote radiosensitivity through targeting CDK1 in hepatocellular carcinoma [25]. miR-1271-5p, sponged by lncRNA UCA1, contributes to apoptosis and inhibits the proliferation capacity in multiple myeloma [26]. Chen et al. indicated that miR-1271 blocks the development of papillary thyroid carcinoma via inhibiting IRS1 expression and inactivating the AKT signaling pathway [27]. Importantly, miR-1271 has been reported as a suppressive factor in OC cells, which suppresses growth and invasion through mediating epithelial-mesenchymal transition [28]. However, the specific molecular mechanism of miR-1271-5p in OC still needed to be detected. In our present study, aberrant miR-1271-5p expression affected the proliferative ability of OC cells. All results identified the inhibitory role of miR-1271-5p in OC cellular behaviors. Downregulation of miR-1271-5p was associated with unfavorable prognosis of OC patients.

miRNAs are enable to regulate physiological and pathological processes through repressing or promoting target mRNAs translation. To further explore the underlying molecular mechanism of miR-1271-5p in OC, we screened the differentially expressed genes according to the TCGA-OC cohort and GTE_X_ database and intersected these upregulated genes with putative target genes of miR-1271-5p. Finally, a common gene TIAM1 was achieved. Here, in this investigation, TIAM1 was identified as a direct target gene of miR-1271-5p in OC for the first time. Consistent with the previous publication, TIAM1 expression was dramatically increased in OC tissues and correlated with outcomes of OC patients. Its expression was negatively regulated by the expression of miR-1271-5p. Furthermore, we also found that TIAM1 played an opposite role with miR-1271-5p in the malignant behaviors of OC cells; TIAM1 overexpression rescued the cell viability, invasion and migration that abolished by miR-1271-5p mimic. In summary, these results depicted that miR-1271-5p/TIAM1 axis may exert an important role in the progression of OC.

Notch signaling pathway plays essential roles in the progression of OC and is active in human OC [29]. Besides, TIAM1 expression positively correlated with the activity of Notch signaling pathway in OC. Additionally, several reports indicate the highly relationship between miRNAs and Notch pathway. For example, the regulatory action of miR-27a, miR-139-5p and miR-433 in different tumors is related with the Notch pathway [30–32]. The epigenetic silencing of miR-199b-5p has also been proven to be linked with the activation of Notch signaling in OC [33]. Thus, to verify whether the biological effect of miR-1271-5p/TIAM1 axis in OC cells is associated with the activity of Notch signaling pathway, we measured the levels of related proteins including Cyclin D1, HES1, Notch and NUMB by western blot. Notch1 is a highly conserved member of Notch family and exerts a vital role in various cancers [34]. Increased Notch1 expression is correlated with unfavorable prognosis of OC patients [35]. HES1 and Cyclin D1 are the downstream genes of Notch1 [36]. NUMB, as a Notch signaling pathway inhibitor, can suppress the activity of Notch signaling via directly binding to the domain of NICD [37]. According to the western blot results, the increased activity of Notch pathway was found in OVCAR-3 cells after miR-1271-5p inhibitor treatment; whereas TIAM1 knockdown inactivated the Notch pathway; co-transfection of miR-1271-5p inhibitor and si-TIAM1 reversed the status of Notch pathway changed by miR-1271-5p inhibitor or si-TIAM1. Similarly, in SK-OV-3 cells, the combination of miR-1271-5p mimic and TIAM1 recovered the activity of Notch pathway to normal status. Collectively, these findings elucidated that miR-1271-5p/TIAM1 axis might regulate the progression of OC through mediating Notch signaling.

However, there are some limitations: due to the shortage of clinical samples downloaded from GEO datasets, we might have magnified the difference of miR-1271-5p expression and its biological function in OC. Therefore, it is necessary to collect the sufficient clinical specimens to validate the suppressive role of miR-1271-5p in the development of OC. In addition, in vivo experiments also required for in-depth validation. Whether Notch signaling pathway is directly or indirectly implicated in the regulatory signaling axis miR-1271-5p/TIAM1 in OC still needs to be evaluated.

In conclusion, the expression of miR-1271-5p is significantly decreased in human OC and down-regulation of miR-1271-5p is associated with poorer outcomes of OC patients. Mechanically, miR-1271-5p represses the proliferation, migration and invasion of OC cells by directly targeting TIAM1 and inactivating the Notch signaling pathway. These observations demonstrate that miR-1271-5p/TIAM1 pair might be developed into a novel therapeutic regimen to better the outcomes and treatment for OC patients.

## Data Availability

The analyzed data sets generated during the study are available from the corresponding author on reasonable request.

## Abbreviations

OC: Ovarian cancer
GEO: Gene Expression Omnibus
TCGA: The Cancer Genome Atlas
CCK-8: Cell counting kit 8
GTEx: Genotype-Tissue Expression
